# Common ELIXIR Service for Researcher Authentication and Authorisation

**DOI:** 10.12688/f1000research.15161.1

**Published:** 2018-08-06

**Authors:** Mikael Linden, Michal Prochazka, Ilkka Lappalainen, Dominik Bucik, Pavel Vyskocil, Martin Kuba, Sami Silén, Peter Belmann, Alexander Sczyrba, Steven Newhouse, Ludek Matyska, Tommi Nyrönen

**Affiliations:** 1 CSC - IT Center for Science, Espoo, Finland; 2 Masaryk University, Brno, Czech Republic; 3 Bielefeld University, Bielefeld, Germany; 4 EMBL-EBI, Hinxton, UK

**Keywords:** authentication, authorisation, IAM, data access, GDPR, GA4GH

## Abstract

A common Authentication and Authorisation Infrastructure (AAI) that would allow single sign-on to services has been identified as a key enabler for European bioinformatics. ELIXIR AAI is an ELIXIR service portfolio for authenticating researchers to ELIXIR services and assisting these services on user privileges during research usage. It relieves the scientific service providers from managing the user identities and authorisation themselves, enables the researcher to have a single set of credentials to all ELIXIR services and supports meeting the requirements imposed by the data protection laws. ELIXIR AAI was launched in late 2016 and is part of the ELIXIR Compute platform portfolio. By the end of 2017 the number of users reached 1000, while the number of relying scientific services was 36.

This paper presents the requirements and design of the ELIXIR AAI and the policies related to its use, and how it can be used for serving some example services, such as document management, social media, data discovery, human data access, cloud compute and training services.

## 1. Introduction

Reliable electronic authentication of users is a requirement to authorise and manage access to the key services and instruments, sensitive data and computing capacities targeted to life science use. Life Science services need to, for example,

1. Identify and group users based on their home organisation (such as the employing institution) information reliably;
2. Manage administrational costs as the number of users and their access rights increase;
3. Prevent poor security practices, such as sharing of user credentials;
4. Ensure access to the data and services is closed as justification, such as the user’s organisational affiliation or other status, changes;
5. Provide separate views on services for users acting in different roles or status (like a bona fide researcher with scientific record of successful research on human data), and
6. Provide an audit trail to comply with privacy laws, including a proper reaction to security incidents.

Increasing the availability of services and data that are subject to access control for scientific, technological and innovation opportunities is a widely-accepted goal in Europe. Within the life-science community this concept is highlighted in management of research consented human data. ELIXIR, the European research infrastructure for biological data, unites the Europe’s leading life-science organisations in managing and safeguarding the massive amounts of data being generated in publicly funded research. It coordinates, integrates, and sustains bioinformatics resources across its member states, and enables users in academia and industry to access vital data, tools, standards, computational and training services for their research. Research Infrastructures (RI), such as ELIXIR, must support federated access to data that needs to be access-controlled.

Technologies in access management are developing rapidly, and new concepts are needed globally to respond to new requirements, including the General Data Protection Regulation ^1^, without creating unnecessary obstacles for research in e.g. human health. In the long term, cross-border access to data resources and services is suggested to lead to beneficial solutions between national and international organisations and their stakeholders.

For example, international collaboration in resource access enables a scalable data infrastructure. Data are going to be described using internationally recognised best practices and standards to enable its pooling. This is crucial for individual reference data set analysis, like the Finnish population genomics ^2^, which is challenging to analyse without access to international reference data and a scalable processing platform.

A number of life-science services must authenticate their users and enforce access and resource policies ^3^. As an outcome, research services have deployed local access management solutions issuing credentials, typically usernames and passwords, for their customers. As a consequence, the users are overloaded by too many credentials they need to remember for login to different services, leading them to adopt poor practices like writing the passwords down or using the same passwords in different services.

Developing and operating access management solutions is expensive, especially advanced services, such as, multi-factor authentication for improved security or user-friendly workflow systems for applying roles and permissions to access resources. These kind of services can be better deployed in a centralised setup where a single access management solution serves several research services (called relying services). Authentication and authorisation is not a core function for research and being able to off-load related functionality would enable the managers of the research services to focus on their core competencies.

In research and education, access management services have been subject of interest since the proliferation of the world-wide web during the latter part of 1990’s. The concept of an authentication and authorisation infrastructure (AAI) was introduced in Switzerland in 2001 as a service layer that provides AAI services for relying parties ^4^. In the commercial sector, the concept of Identity and Access Management (IAM) has later emerged with similar thematic focus. Standards (such as SAML), open source products (such as Shibboleth and SimpleSAMLphp) and services (most importantly, research and education identity federations ^5^) have all been established during the 2000’s, enabling relying services to make use of the researchers’ home university accounts for login.

The ELIXIR AAI development started in 2014 as one of the ELIXIR Compute platform key components based on a generic use cases that cover most ELIXIR services. ELIXIR AAI intends to serve the needs of the various ELIXIR communities’ services operated by the ELIXIR Nodes in different countries. Some of the communities (such as the plant and marine) have moderate security needs whereas others (such as the sensitive human data and the rare disease) have strict requirements for authentication and authorisation of the researchers.

This paper describes the key concepts of ELIXIR AAI and its uptake in the community during the first two years of operation. The work describes the requirements the authors have collected for the ELIXIR AAI based on use case analysis, introduces the ELIXIR AAI design to meet the requirements, provides an overview of the ELIXIR AAI policies to complement the design, and describes technology use by selected early adopters. ELIXIR AAI uptake is advancing rapidly. Therefore, our future work will focus on the sustainable expansion of the services into the broader life science service provider community.

## 2. Requirements on ELIXIR AAI

This section presents the requirements that have driven the design of the ELIXIR AAI. The requirements were drawn from the analysis of the use cases collected in autumn 2014.

### 2.1. ELIXIR identity and linked external accounts

Researchers within Europe and beyond can register an ELIXIR identity to access the relying services. The relying services identify each user by an ELIXIR identifier which is an opaque non-reassignable string. When a human-readable presentation of the ELIXIR identity is needed for instance in the user interface, a parallel ELIXIR username can be used instead. The ELIXIR identifier and the ELIXIR username are exemplified in Table 1 below.

**Table 1.** Examples on ELIXIR identifier and username.

ELIXIR identifier	28c5353b8bb34984a8bd4169ba94c606@elixir-europe.org
ELIXIR username	philip@elixir-europe.org

The intention is that a person registers one ELIXIR identity and uses that throughout their career, just updating their affiliation information (see the next section) on an affiliation change. Although there is no practical means to prevent researchers from registering several ELIXIR identities, it is believed that having several ELIXIR identities would be confusing especially for the users themselves.

There is no password associated to an ELIXIR identity. Instead, the registration process requires the user to link to one or more of their existing academic or commercial accounts which are then used for login. Following external accounts can be associated to an ELIXIR identity:

- Researcher’s account in their home university or research institution provided it has an Identity Provider server available via the eduGAIN interfederation service. This approach is preferred because the home university is able to deliver also the fresh affiliation information for a researcher
- ORCID identity as provided the ORCID community
- The commercial identity providers Google and LinkedIn.

For accountability reasons, the ELIXIR identity represents a single natural person. ELIXIR AAI does not accept shared accounts or accounts representing a specific role (e.g. a position like “on-duty operations manager” that circulates among a pool of persons). This is excluded in the Acceptable Usage Policy that the users need to commit to when they register an ELIXIR identity and, when possible, by technical checks on the external accounts.

### 2.2. Attributes associated to an ELIXIR identity

ELIXIR AAI enriches the ELIXIR identities with extra attributes to serve the authorisation needs of the relying services. This section describes the key attributes.

***2.2.1. User’s home organisation(s).*** In the ELIXIR research infrastructure, users are typically affiliated with an organisation (called a home organisation), such as, a university, research institution or a private company and the permissions to access services is often coupled to their continuing affiliation. The current affiliation attribute is a multivalued attribute that indicates the organisation(s) the end user is currently affiliated with and the type of the affiliation. A user can have several home organisations at the same time if they for example work or study in several organisations in parallel. The relying services who decide the user’s access rights based on their affiliation can monitor changes in the attribute.

A user can have three types of affiliations with their home organisation as described in Table 2 below. *Faculty* affiliation type is believed to most accurately represent the person’s role as a researcher in the home organisation, although the exact definition leaves some institutional freedom. *Member* affiliation covers also other kind of Home organisation members, such as staff and students and *affiliate* the rest. The syntax and semantic of the attribute follows the eduPersonScopedAffiliation attribute defined in eduPerson schema ^6^.

**Table 2.** Home Organisation affiliation attribute’s types, their semantics and registration procedures.

Affiliation type	Semantics	Procedure to register a value in ELIXIR authentication and authorisation infrastructure (AAI)
Faculty	The person is a researcher or teacher in their home organisation.	• Perform login using the home organisation Identity Provider where the home organisation releases “faculty” attribute, or • be assigned the value by trusted person in their home organisation
Member	"Member" is intended to include faculty, staff, student, and other persons with a full set of basic privileges that go with membership in the home organisation, as defined in eduPerson ^6^.	As above
Affiliate	The "affiliate" value indicates that the holder has some definable affiliation to the home organisation not captured by faculty and/or member ^6^.	As above, or • Demonstrate to control an e-mail address that belongs to the home organisation

The preferred way to populate the affiliation attribute is to retrieve it programmatically from the home organisation using the eduGAIN service (see section 2.1). There are also other ways described in the table for those home organisations who do not support programmatic access. The validity of the attribute values is guaranteed by asking the user to refresh the value every 12 months using the procedure described above.

***2.2.2. Group membership.*** In many relying services, users’ access rights are coupled to their membership in a group associated with the service. For instance, if a project is assigned a resource quota in a computing cluster, any member of the project group should be able to log in and initiate computations. In that scenario, the resource quota would be assigned to a group and any member of the group can consume the shared resource allocation.

ELIXIR AAI has a service for managing ELIXIR users’ group memberships and roles in the groups they belong to. Management of groups is done using a web interface or an API.

Each user can belong to one or multiple groups simultaneously. This is represented by the user having a “member” role in the group. A group member can have also arbitrary extra roles in the group, such as “secretary” or “chair”.

Each group can have one or several owners (members with a special role “owner”). The creator of a group becomes the initial owner of the group. Owners are able to

- delegate group ownership to other members or groups
- manage the group’s properties (such as name)
- invite group members (requires confirmation by the invited user)
- add group members (no confirmation needed by the invited user)
- remove group members
- assign and delete additional attributes (roles) for users in the group
- define and manage registration form and process for the group

### 2.3. Tiered access to datasets

The Global Alliance for Genomics and Health is promoting a tiered access to sensitive human data, which consists of three access levels: public access for data with no access control needs, and registered as well as controlled access levels for data whose access is based on the users’ roles and entitlements.

ELIXIR AAI has services that support implementation of the registered and controlled access. These services support the dataset owner’s also in meeting their obligations originating from the General data protection regulation, including security by design, accountability and implementation of appropriate technical and organisational measures to secure the data in the registered and controlled access tier.

***2.3.1. Controlled access data.*** ELIXIR AAI has a service that researchers can use for applying for access rights to sensitive datasets and the dataset owners to approve or rejects the data access applications. The Principal Investigator of a project fills in a data access application on behalf of all project members and commits to the dataset’s license terms. The electronic application is then circulated to the Data Access Committee nominated by the dataset owner as defined in the workflow of the dataset. Once approved, the user’s entitlements to access the datasets are presented to the data archive or computing environment for access control enforcement. The dataset owners relying on the system can dynamically revoke and audit the users’ data access rights. Requirements of the service are presented in detail in 7.

***2.3.2. Registered access data.*** The process described above for the controlled access datasets is laboursome both for the applicant and the dataset owners and the application review process may introduce an unnecessary delay to the researcher’s data access.

To reduce the administrative burden, the Global Alliance for Genomics and Health has proposed that datasets whose privacy impact is limited could deploy a more light-weight schema where the researcher needs to just demonstrate they are a researcher in good standing and commit to the general terms of the registered access. Once done, the researchers could access any registered access datasets or services without further applications. An example of such services are the ELIXIR Beacons ^8^. The requirements of registered access are further described in 9.

ELIXIR AAI supports registered access by providing a mechanism for the researcher to demonstrate their bona fide status and make the necessary attestations to confirm that they are committed to the terms of registered access data. As a result, the users’ ELIXIR identity is amended with an extra attribute declaring that they have a status as a bona fide researcher.

### 2.4. Step-up authentication

Some of the relying services, especially those related to sensitive human data, expect not just fine-grained authorisation but also authentication which relies on multiple authentication factors (something you know, something you have, something you are).

Authentication to ELIXIR AAI is carried out by the external authentication providers the user has linked to their ELIXIR identities. Typically, those providers offer just password based authentication which is, despite its wide use, vulnerable to various attacks.

ELIXIR AAI offers a step-up authentication service for multi-factor authentication. The user is first authenticated by their external authentication provider and subsequently by a second authentication factor delivered by ELIXIR AAI.

### 2.5. Interfaces for relying services

***2.5.1. Authentication.*** Towards the relying services, ELIXIR AAI supports SAML 2.0 and OpenID Connect protocols for user authentication and delivery of the attributes described above. The relying service can also use a mechanism to request a step-up authentication.

Although OpenID Connect and its underlying OAuth2 protocol can be extended also to non-web services, such as native apps, they are mostly limited to an environment where the user has a web browser to access or launch the service. Services such as data transfer may require authentication using X.509 certificates. ELIXIR AAI also supports credential translation where a user can receive a X.509 certificate after a successful authentication on ELIXIR AAI.

***2.5.2. Attribute push.*** In the basic scenario, the Relying party receives the necessary attributes from ELIXIR AAI using the SAML 2.0 or OpenID Connect protocols when the user logs in as described above. However, some services need a batch synchronisation of users’ attributes although they do not log in to the service. Examples of such services are mailing list services (becoming a group member subscribes them to an associated mailing list service) or IaaS cloud middleware (closing a project shuts down all associated virtual machines).

ELIXIR AAI serves those Relying services with batch based attribute synchronisation when needed.

## 3. ELIXIR AAI design

This section describes the design of the ELIXIR AAI that covers the requirements presented in the previous section. All the components are implemented using open source software.

### 3.1. Overview

**Figure 1.**
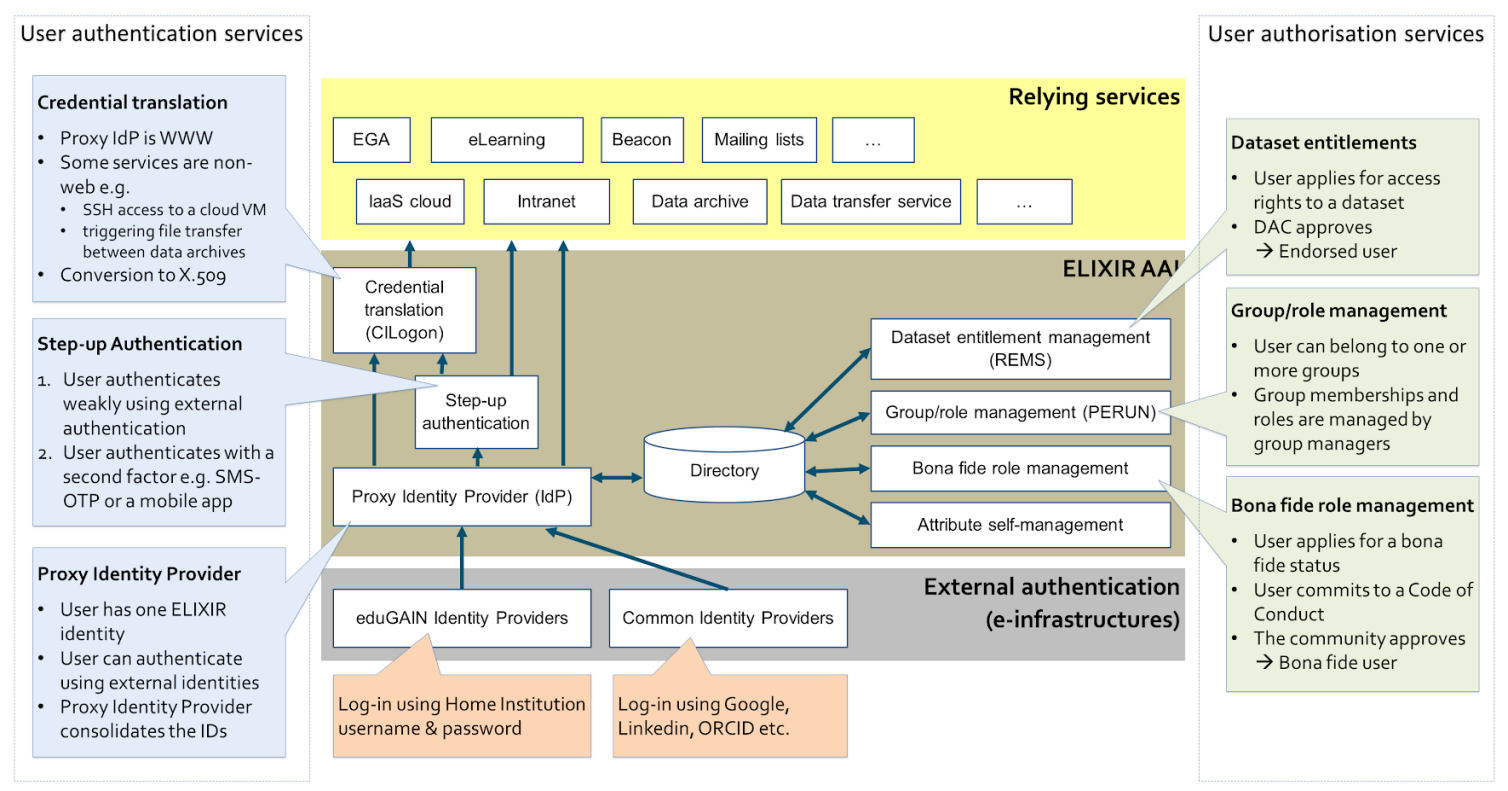
ELIXIR authentication and authorisation infrastructure (AAI) design overview.

Figure 1 below presents an overview of the ELIXIR AAI services. The components are described in detail in the next sections.

In the upper part of the figure are the services relying on the ELIXIR AAI for authentication and authorisation of their users. ELIXIR AAI can support simple tools and services such as the wikis or mailing lists with moderate authentication and authorisation requirements, as well as more complex relying services, such as archives of sensitive data, scientific workflow systems and IaaS clouds for processing the data.

The external authentication providers are down in the figure. eduGAIN, Google, ORCID and Linkedin are supported. A user can decide which one to use during login process.

The middle part of the figure presents the ELIXIR AAI service components. The ELIXIR AAI services for authentication are in the left hand side. The ELIXIR AAI services for decorating the authenticated identities with extra attributes for authorisation are in the right hand side.

### 3.2. ELIXIR proxy identity provider

When a user has authenticated using an external authentication provider they first land on the ELIXIR proxy identity provider (IdP). The proxy IdP acts as a relying service towards the external authentication providers and as an Identity Provider towards the relying services and other AAI components. If the proxy IdP observes a new user it triggers automatically a user registration sequence during which the ELIXIR identifiers (see section 2.1) are assigned to them.

The proxy IdP together with the Perun system ^10^ is responsible for account linking, which tries to avoid a user registering multiple ELIXIR identities by performing heuristics. When the system observes similarity between an existing and a new user account it proposes the user to link the external account to their existing ELIXIR identity, instead. The linking is done by asking the user to log in using the new account and, subsequently, using the existing account.

The proxy IdP is based on the SimpleSAMLphp product and is replicated geographically for high availability. The OpenID Connect integration to relying services is done using the MITREid product.

### 3.3. Step-up authentication

If the relying service requires multi-factor authentication, the user will be redirected to the step-up authentication service that performs a subsequent extra authentication using a second authentication factor.

In the current deployment, the step-up authentication relies on the Time-based One-Time Password (TOTP) standard ^11^ and a smartphone app that the user needs to install in their smartphone and register to ELIXIR AAI. Once registered, the TOTP app delivers a 6 digit one-time password that is valid for one minute. The user needs to activate their smartphone app and type the one-time password the app gives to the Step-up authentication service’s web page before it expires.

The registration of the second authentication factor to the correct ELIXIR identity is based on delivering an SMS to the user’s registered cellphone number. ELIXIR is deploying a process to record the ELIXIR identity holder’s trusted cellphone number.

The service is based on a TOTP server side implementation made at ELIXIR-Finland ^12^. In their smartphones the users can use any standard TOTP app available in the appstores, such as, FreeOTP or Google authenticator.

### 3.4. Credential translation

The credential translation component currently supports delivering X.509 certificates to the users. The component asks the user to log in using the Proxy IdP and issues them a certificate. The deployment is based on the CILogon software and RCAuth service pilot delivered by the AARC project and is currently operated by NIKHEF.

The X.509 issued credentials are currently used for gridFTP and access to some IaaS cloud middleware services.

### 3.5. ELIXIR directory and attribute self-management

ELIXIR directory is an abstract container for storing all ELIXIR identities and attributes. It serves as a common integration point for services that manage user, group and service attributes and authorisations in ELIXIR AAI. It is a service component internal to ELIXIR AAI, however it has API which can be used by relying services to obtain additional data online.

Access to all the data stored in ELIXIR directory is controlled. Majority of ELIXIR directory services is managed by Perun ^10^ system.

A user can self-manage some of their attributes in the ELIXIR directory, such as their e-mail address. ELIXIR AAI provides the users also a control panel where they can manage the external authentication providers linked to their ELIXIR identity and review all the data stored about them in ELIXIR directory.

### 3.7. Bona fide role management service

In the bona fide role management service the user can register their bona fide status and make the attestations as described in section 2. The bona fide status is then stored as an extra attribute for the user’s ELIXIR identity and is available for the relying services.

In the current implementation, the user can demonstrate their researcher status by

- Acquiring a “faculty” attribute from their home organisation (see section 2.2.1), or
- Asking another ELIXIR user who has a “faculty” status in their home organisation to vouch for their bona fide status, or
- matching their ELIXIR identity against the European PubMed archive to demonstrate they are an author of at least one publication.

The bona fide management service also asks the user to make the attestations ^9^ that are necessary to receive the bona fide status. If several parallel attestations consumed by different relying services emerge, they will be supported, too.

The functionality is implemented using the REMS ^7^ and Perun ^10^ software.

### 3.8. Group management service

Using the group management service of ELIXIR AAI a user can form new groups and manage their members. The groups can be hierarchical and the management of the subgroups can be delegated to other users or groups. Each group can have its own registration process defined. Managers of a group can then gather additional data from users or control how the users are invited into the group. The ELIXIR AAI can expose the group memberships to relying services via various channels like SAML attributes, OpenID Connect claims or directly push them to the service.

ELIXIR is currently using the group membership e.g. for managing membership in the projects and related mailing lists to build the ELIXIR services. The group memberships are also consumed by IaaS clouds integrated to ELIXIR. The group membership service is provided by the Perun ^10^ software.

### 3.9. Dataset authorisation management service

Relying services can register their datasets to the dataset authorisation service and define application forms and related workflows for managing the dataset access rights. The ELIXIR user can then apply for access rights to a dataset and the application is circulated to the related Data Access Committee for approval. Once approved, the ELIXIR AAI uses OAuth2 protocol to expose the access rights to the relying service for access control enforcement.

The Dataset authorisation service is implemented using the REMS software ^7^.

## 4. ELIXIR AAI policies

The technical infrastructure introduced in the previous section is complemented by a set of policies relevant for use of the AAI. The policies described below cover the expectations on the behaviour of the end users, relying services and AAI operators.

### 4.1. Policies for end users

When a user registers an ELIXIR identity they must commit to ELIXIR AAI’s Acceptable Usage Policy (AUP) ^13^. The AUP regulates how and for which purposes the relying services can be used and how the authentication credentials must be protected and used. The AUP also informs the user on the processing of their personal data, including the ELIXIR AAI log files.

The AUP intends to set the basic level of expectations on the behaviour of the users in the relying services and allows ELIXIR to exclude misbehaving users. Individual relying services can complement the AUP by their own terms of service, based on their specific needs.

### 4.2. Policies for relying services

The relying services of ELIXIR AAI must support life science research. This requirement allows also services beyond ELIXIR research infrastructure to integrate to the ELIXIR AAI, as long as the services benefit life science research. This is desirable because there are also e-infrastructures and other research infrastructures serving the ELIXIR community.

In order to integrate to ELIXIR AAI, the relying services need to respect the applicable laws, including the European data protection laws. If the service is established outside the European Economic Area and countries which guarantee adequate protection of personal data, the relying service must guarantee it takes appropriate safeguards to protect the ELIXIR identities it receives from ELIXIR AAI.

### 4.3. Policies for AAI operators

ELIXIR AAI is operated by the Czech and Finnish ELIXIR Nodes. The ELIXIR AAI policy obligates the AAI operators to take care of the information security and availability of the AAI services professionally. Normally the AAI services are expected to be available 24-by-7.

The AAI operators must use the personal data only for what is needed for the operations of the AAI. Log files must be used only for administrative, operational, accounting, monitoring and security purposes. The operators can provide statistics on the use of the AAI service but the statistics must not reveal individual users.

## 5. Uptake and outreach

ELIXIR AAI became operational in November 2016. It is a service that belongs to the Compute platform of ELIXIR together with the cloud and data transfer services. In the end of 2017, ELIXIR AAI had 1097 users who belong to 124 groups and could log in to 36 relying services. Additionally, 71 relying services were testing their integration to the ELIXIR AAI, including major European e-Infrastructures EGI and EUDAT. An average of 3000 logins were observed in a month.

Early relying parties of the ELIXIR AAI include internal project management (e.g. Intranet), social media applications (e.g. Virtual coffee room), request tracking systems (e.g. RT or TOPdesk), data discovery (e.g. Beacon), data and sample access (e.g. THL biobank), and cloud computing service (e.g. EGI fedCloud, EMBL-EBI Embassy and de.NBI cloud). This section provides examples on the use of ELIXIR AAI in these relying services to manage users and access rights gives an overview of the possibilities.

### 5.1. Implementation to document management

ELIXIR Intranet is an internal document management service provided from the central ELIXIR Hub to manage documentation of the distributed ELIXIR infrastructure. The intranet relies on ELIXIR AAI for the user and group management. Experts belonging to a particular group like Heads of Nodes or Technical coordination have different access and visibility on the ELIXIR documentation.

### 5.2. Implementation to social media application

ELIXIR Virtual Coffee Room is a service run by the Estonian ELIXIR Node to enable experts from ELIXIR communities to interact online. ELIXIR AAI is implemented in the user and group management. For instance, the training and technical experts from ELIXIR Nodes belong to an interest group with their own dedicated group spaces where they can virtually meet even though they are working across the Nodes.

### 5.3. Implementation to data discovery service

ELIXIR Beacons ^8^ are part of the global Beacon network and a GA4GH driver project influencing many of the emerging global standards that ensure interoperability for life-science researchers. The ELIXIR Beacons enable discovery of genomic datasets using a specialised query interface. Once a dataset has been discovered, ELIXIR AAI allows for the user to gain data access rights if they are recognised as a bona fide researcher to access registered access data. Eventually, the data discovery can lead the user to apply for controlled access rights from the data owners.

### 5.4. Implementation to human data and sample access

National Institute of Health and Welfare THL biobank is part of BBMRI and linked to ELIXIR Finland. THL uses ELIXIR AAI REMS to manage access application to biobank samples from the Finnish population-level cohorts, and datasets created from the samples. THL was the first sensitive data controller in Europe to federate data access authorisations electronically in collaboration with ELIXIR. Electronic data access entitlements coupled to the reliable identification users is part of the national strategy of Finland to comply with the General data protection regulation.

### 5.5. Implementation to cloud service

ELIXIR Germany’s de.NBI cloud and EMBL-EBI’s Embassy cloud trust ELIXIR AAI to manage user access to their cloud portal. The de.NBI cloud works as a technical integration point between compute and data resources for bioinformatics in Germany. The cloud access authorisation decisions are made by the local compute providers, and the resource allocation is recorded in ELIXIR AAI and pushed to the cloud centers. In the EMBL-EBI Embassy, user access attributes are released from ELIXIR AAI to the cloud provider when a user logs in to the cloud middleware.

### 5.6. ELIXIR training and AAI

The ELIXIR training portal TeSS relies on ELIXIR AAI, and the ELIXIR AAI team has organised three training events to help the potential relying services to integrate to ELIXIR, including providing ELIXIR AAI training material contents in TeSS. The hands on trainings have focused on helping the participants to install a SAML Service Provider or OpenID Connect Relying Party and configure it to rely on ELIXIR AAI for user authentication and consumption of user attributes in their services.

Further information and relying service integration instructions are available here. Statistics on the number of logins to relying services is available here.

## 6. Future work

ELIXIR is one of the 13 life science research infrastructure recognised by ESFRI 2016 roadmap ^14^. Although the infrastructures are different in their focus and security requirements, many of them share the need for authenticating and authorising their end users. Many of the researchers are also using services from several infrastructures which would be made simpler by a common AAI.

Since 2016, ELIXIR has worked together with the other infrastructures in CORBEL, the ESFRI cluster project for life sciences. CORBEL has collected AAI use cases from the participating research infrastructures and compiled them to a requirements specification for a common Life Science AAI ^15^. The requirements specification has been used as the basis for a Life Science AAI pilot in the context of AARC2 project. Funding has been applied for deploying the Life Science AAI into production.

The Global Alliance for Genomics and Health (GA4GH) is a policy-framing and technical standards-setting organization, seeking to enable responsible genomic data sharing within a human rights framework. Its data use & researcher identities (DURI) workstream facilitates and enables the harmonisation of researcher identities by defining who is a bona fide researcher and one or more identity providers that respects this definition and can provide a portable electronic identity. Compatibility with the DURI deliverables, such as the library card concept ^16^, is a future work item for ELIXIR AAI.

## 7. Conclusions

ELIXIR has developed a comprehensive AAI service portfolio for authentication of researchers and assisting the relying services to decide what the researchers are permitted to do in the service. The service belongs to the ELIXIR Compute platform and is deployed using open source components. The ELIXIR AAI is available for relying services that support life science research.

This paper described the requirements on the AAI service and how they have been compiled to a design of the ELIXIR AAI. The related policies were shortly described to complement the technical perspectives. The ELIXIR AAI has been in production since late 2016 and ELIXIR is inviting new services to integrate to it.

## Data availability

No data are associated with this article

## Software availability

The ELIXIR AAI homepage with guides of how to connect to service and as well as policy documents is available here: https://www.elixir-europe.org/services/compute/aai

CESNET/Perun

Source code available from: https://github.com/CESNET/perun/tree/v3.1.0

Archieved source code at time of publication: http://doi.org/10.5281/zenodo.1308874 ^17^

CESNET/Perun-mitreid

Source code available from: https://github.com/CESNET/perun-mitreid/tree/v1.11.0

Archieved source code at time of publication: http://doi.org/10.5281/zenodo.1299810 ^18^

CESNET/Perun-services

Source code available from: https://github.com/CESNET/perun-services/tree/v3.1.0

Archieved source code at time of publication: http://doi.org/10.5281/zenodo.1300300 ^19^

CESNET/Perun-simplesamlphp-module

Source code available from: https://github.com/CESNET/perun-simplesamlphp-module/tree/elixir

Archieved source code at time of publication: http://doi.org/10.5281/zenodo.1300769 ^20^

CESNET/Perunauthorize-simplesamlphp-module

Source code available from: https://github.com/CESNET/perunauthorize-simplesamlphp-module/tree/v1.0.0

Archieved source code at time of publication: http://doi.org/10.5281/zenodo.1300765 ^21^

CESNET/Proxystatistics-simplesamlphp-module

Source code available from: https://github.com/CESNET/proxystatistics-simplesamlphp-module/tree/v1.1.0

Archieved source code at time of publication: http://doi.org/10.5281/zenodo.1300761 ^22^

CESNET/Simplesamlphp

Source code available from: https://github.com/CESNET/simplesamlphp/tree/v1.15.4

Archieved source code at time of publication: http://doi.org/10.5281/zenodo.1298400 ^23^

CSC/Rems

Source code available from: https://github.com/CSCfi/rems/tree/v2.1

Archieved source code at time of publication: http://doi.org/10.5281/zenodo.1297336 ^24^

CESNET/Perun-mitreid is licenced under the Apache-2.0 licence, other components implemented by CESNET under the BSD-2-clause licence and the component by CSC under the MIT licence.
