# Supplementary material for: Common ELIXIR Service for Researcher Authentication and Authorisation
Source: F1000Res. 2018 Aug 6;7:ELIXIR-1199. [Version 1] doi: 10.12688/f1000research.15161.1 (PMC6124379; doi:10.12688/f1000research.15161.1)
Supplement: Peer review history

# Peer review: Referee response for version 1

**DOI:** 10.5256/f1000research.16516.r36862

**Published:** 2018-09-06

**Authors:** Marcus Hardt

**Affiliations:** 1 Steinbuch Centre for Computing (SCC), Karlsruhe Institut of Technology (KIT), Karlsruhe, Germany

The article provides an overview on the ELIXIR AAI. It is written on a very high level of abstraction.  

 The described system integrates services deployed in many different countries and organisations on one side with federated users on the other side. The requirements that lead to developing the system are outlined, all the important technical components are described and justified with respect to the requirements. The section about the policies that describes the duties of the involved parties and their legal / organisational interplay amongst each other is very short, and therefore incomplete.  In the uptake section the authors prove their success with the number of registered users and the impressive number of available services.

 The strength of this article is its high level of abstraction. This allows non-technical people to get an overview of the developed method. The downside of this is that many technically hard points that had to be solved were not able to be mentioned.

 From my perspective a few points deserve additional work:

- Policies: You are following the AARC policy starter pack. I think the article would benefit, if you  even if briefly - mentioned all the relevant policies in the field.
- Trust: You mention services that accept users authenticated elsewhere in the world. How was it possible to convince these services to trust other identity providers and/or group-managers in foreign countries that a given user is in fact the one he claims to be.
- Finally, a lessons learned section will help others to reproduce the presented method.

I have read this submission. I believe that I have an appropriate level of expertise to confirm that it is of an acceptable scientific standard.

# Peer review: Referee response for version 1

**DOI:** 10.5256/f1000research.16516.r36860

**Published:** 2018-08-31

**Authors:** Nicolas Liampotis

**Affiliations:** 1 GRNET SA, Glyka Nera, Greece

This paper describes the ELIXIR Authentication and Authorisation Infrastructure (AAI) which enables researchers to access ELIXIR services and resources using a single set of credentials. 

 The authors start by presenting the requirements extracted from the analysis of the use cases that were collected during the design phase of the ELIXIR AAI. The different requirements are described in detail and cover aspects related to the ELIXIR identity and its attributes, the authorised access to data, the need for stepping up the authentication strength via multi-factor authentication and the interfacing with service providers based on standard technologies such as SAML and OpenID Connect.

 The authors then describe the key features of the ELIXIR AAI design that was driven from the analysed requirements. Following the presentation of the design, the paper discusses the set of policies pertaining to the end users, relying services and AAI operators.

 The paper also provides information related to the uptake of the ELIXIR AAI during its first two years of operation in production.

 The inclusion of links to the ELIXIR AAI documentation and the relevant software repositories is particularly useful for implementers/operators of AAI solutions for research collaboration.

 Please consider the following edits:

- Page 4. "Instead, the registration process requires the user to link **to** one": remove **"to"**
- Page 4. ORCID identity as provided * the ORCID community: insert **"by"**

I have read this submission. I believe that I have an appropriate level of expertise to confirm that it is of an acceptable scientific standard.

# Peer review: Referee response for version 1

**DOI:** 10.5256/f1000research.16516.r36861

**Published:** 2018-08-22

**Authors:** Dixie Baker

**Affiliations:** 1 Martin, Blanck and Associates, Alexandria, VA, USA; 2 Global Alliance for Genomics and Health, Alexandria, VA, USA

Given the broad, multinational applicability and utility of the ELIXIR AAI, this article is of high value, and I recommend its acceptance with some modifications.  

 Regarding explanation of the "method," although Section 2 is titled "Requirements for ELIXIR AAI," which begins "This section presents the requirements that have driven the design of the ELIXIR AAI," in actuality, no "requirements" are articulated.  Instead, the section describes the ELIXIR AAI system as implemented, so the reader is left to surmise what the "requirements" might be.  Also, as described in section 2.1 and reinforced throughout, ELIXIR seems more akin to a broker of identities authenticated by multiple IdPs than an identity provider.  

 With respect to providing sufficient detail to enable replication, without clearly articulated requirements,  it would be difficult for anyone to replicate this implementation.  For example, Section 2.3 (within the "Requirements" section begins by explaining that the Global Alliance for Genomics and Health is "promoting a tiered access to sensitive human data..."  Does this mean that anyone attempting to replicate this work must join with GA4GH in promoting this access model?  

 Regarding whether the "conclusions about the method and its performance adequately supported by the findings," this article does not present any conclusions or performance data.

I have read this submission. I believe that I have an appropriate level of expertise to confirm that it is of an acceptable scientific standard, however I have significant reservations, as outlined above.

## References

[1] LindenMProchazkaMLappalainenIBucikDVyskocilPKubaMSilénSBelmannPSczyrbaANewhouseSMatyskaLNyrönenT: Common ELIXIR Service for Researcher Authentication and Authorisation. *F1000Research*.2018;7: 10.12688/f1000research.15161.1 10.12688/f1000research.15161.1 PMC612437930254736

# Peer review: Referee response for version 1

**DOI:** 10.5256/f1000research.16516.r36859

**Published:** 2018-08-20

**Authors:** Bengt Persson

**Affiliations:** 1 Department of Molecular Biology, Science for Life Laboratory, Uppsala University, Uppsala, Sweden; 2 Department of Medical Biochemistry and Biophysics, Science for Life Laboratory, Karolinska Institutet, Stockholm, Sweden

This is a well-written article giving a clear presentation of the ELIXIR infrastructure for researcher authentication and authorisation (AAI). The AAI service enables for a life science researcher to obtain an ELIXIR identity that can be used for getting access to restricted ELIXIR services. The authors describe the AAI service systematically and thoroughly, including the rationale behind and the initial requirements, the AAI design and the different components of the system, AAI policies, and examples of applications. Links to software repositories and appropriate references are provided. In conclusion, this is an excellent contribution to the F1000Research.

I have read this submission. I believe that I have an appropriate level of expertise to confirm that it is of an acceptable scientific standard.
